# Seasonal variations in the phenolic profile, antioxidant activity, and mineral content of south Indian black tea (*Camellia sinensis* (L.) O. Kuntze)

**DOI:** 10.1038/s41598-023-45711-1

**Published:** 2023-10-31

**Authors:** Kottur Govindasamy, Daisylin Anbu Sujitha Sugumar, N. Mani Kandan, N. Nagaprasad, Krishnaraj Ramaswamy

**Affiliations:** 1 Department of Chemistry, P.A. College of Engineering, Pollachi, Coimbatore, Tamilnadu 642 002 India; 2 Department of Chemistry, Sri Sai Ram Institute of Technology, West Tambaram, Chennai, Tamilnadu 600 044 India; 3 Department of Mechanical Engineering, P.A. College of Engineering and Technology, Pollachi, Coimbatore, 642002 India; 4 Department of Mechanical Engineering, ULTRA College of Engineering and Technology, Madurai, Tamilnadu 625104 India; 5 Centre for Excellence-Indigenous Knowledge, Innovative Technology Transfer and Entrepreneurship, Dambi Dollo University, Dambi Dollo, Ethiopia; 6 Department of Mechanical Engineering, College of Engineering and Technology, Dambi Dollo University, Dambi Dollo, Ethiopia

**Keywords:** Energy science and technology, Engineering, Materials science, Mathematics and computing

## Abstract

In the Anamallais region of south India, crop shoots from the UPASI-3, UPASI-9, UPASI-17, Assam seedlings, and TRI-2043 cultivars were examined for seasonal variations in total phenolics, antioxidant activity, and minerals during four harvest seasons: summer (January to March), premonsoon (April and May), monsoon (June to September), and winter (October to December) of two consecutive years. The total phenolics of all cultivars were lower in monsoon period and grew over rest of the seasons and it was greater during summer. Crop shoot antioxidant activity as measured by the DPPH radical scavenging experiment exhibited a similar pattern to total phenolics. Summer was the season with the highest antioxidant activity across all cultivars, followed by premonsoon, winter, and monsoon. On the other hand, the employed cultivars differed noticeably in terms of seasonal change of minerals. These results appear to indicate that the harvest period is hypercritical in deciding the antioxidant potency of tea crop shoots.

## Introduction

Millions of people all around the world drink tea, which is one of the most popular non-alcoholic and nutritious beverages, from dawn till sunset. High-quality tea shoots are produced by healthy tea plants, and these shoots differ depending on the tea cultivars and environmental factors including soil type, altitude, and climate of the plant's growing region. One of India's most significant agricultural, environmentally friendly, labour-intensive, employment-generating, and export-focused industries is tea^1^. Tea, in addition to being a beverage crop, has various medical qualities due to its lack of calories and the inclusion of several organic biochemicals such as flavanols, amino acids, and vitamins. Tea's nutritional and therapeutic value stems from its unique blend of ingredients such as proteins, carbohydrates, amino acids, lipids, vitamins, minerals, alkaloids, and polyphenols^2^. It is a natural antioxidative agent capable of reducing a wide variety of ailments such as cancer and heart disease due to the presence of polyphenols and catechins^3^. As a result, tea appears to be a potent chemo preventive agent against toxic substances, free radicals, and carcinogens^4^. Tea also provides essential minerals and trace components for human health such as K, Mn, Cr, Ni, and Zn. Tea drinking adds to the daily dietary needs of various essential elements and may be a source of manganese and a large amount of potassium, which may be useful to hypertensive patients^5^.

Finding plants with high antioxidant capacities has become more and more important recently because they can shield people from free radicals and slow the onset of many chronic diseases^6^. Young tea shoots have a dry weight basis content of greater than 35% polyphenols. Unfermented green tea contains more flavanols and phenolic acids than black tea, with caffeic acid, gallic acid, and coumaric acid being the most prevalent ones. It is well known that one of the key elements affecting the tea's general quality is the phenolic content of young shoots.

Several tea researchers revealed the results of the study on the chemical makeup of tea shoots^7,8^. However, the diversity in the chemical composition of tea shoots at various harvest times in south India has not yet been documented. The current study aims to evaluate seasonal fluctuations in total phenolics, antioxidant activity, and minerals in young tea shoots of popular five tea cultivars in south India. This information will be valuable in pinpointing the ideal time of harvest for obtaining high antioxidant characteristics.

## Material and methods

### Experimental site and sampling

The study was carried out in Valparai, Tamil Nadu, India, at 1050 m above MSL in the TRI (Tea Research Experimental Farm) of the UPASI (United Planters' Association of Southern India). Three cultivars—UPASI-3, UPASI-9, and UPASI-17—that stand in for "Assam," "China," and "Cambod" were employed as test subjects, together with seedlings of "Assam" seedling and TRI-2043, a mutant created by the Tea Research Institute in Sri Lanka. Sampling was done regularly during a two-year period at intervals of one month. The study area received annual rainfall of 3829.3 mm and has four dissimilar seasons (Summer, Pre-monsoon, Monsoon and Winter), with an average temperature of 28.2 ◦C (max.) and 9.1 ◦C (min.) The average meteorological data that were collected over this time are displayed in Table 1 below. The plant we have used in this report was cultivated in Pollachi, Tamilnadu, India. This study complies with relevant legislation and international, national, and institutional guidelines. To collect the plant permissions were obtained P.A. College of Engineering, Research department, Pollachi, Coimbatore-642 002, Tamilnadu, India.

**Table 1:** Meteorological data of experimental farm.

Month	Temperature (°C)		Relative humidity (%)		Rain fall (mm)	Mean sun shine period (h/day)
	Mean min	Mean max	8.30 a.m	2.30 p.m		
January	10.6	26.3	87.0	56.0	10.8	7.08
February	9.1	28.2	85.0	44.0	7.6	7.57
March	14.4	27.6	87.0	66.0	157.8	6.43
April	16.4	27.1	87.0	77.0	132.2	6.17
May	17.6	26.2	90.0	82.0	523.1	4.08
June	16.9	23.9	90.0	83.0	538.4	2.53
July	17.6	22.2	95.0	88.0	859.6	1.10
August	16.8	23.3	91.0	84.0	526.2	2.55
September	16.9	23.2	91.0	87.0	536.0	2.23
October	16.7	24.5	91.0	87.0	278.4	2.40
November	15.5	25.2	89.0	84.0	259.2	3.47
December	11.0 Total	26.7	77.0	63.0	0.0 3829.3	7.05

### Preparation of alcoholic extract for analysis of polyphenols and catechin

Tea shoots comprising three leaves and an apical bud (about 1 g) were ground well with 100% ethyl alcohol. The contents were filtered and the filtrate was made up to 50 mL with ethyl alcohol. The alcoholic extract was used for estimation of polyphenols and catechins.

### Estimation of polyphenols

One mL of the alcoholic extract was diluted to 50 mL with distilled water. Two mL of diluted extract was added with 4.0 mL of Folin-Ciocalteu’s reagent (1:1) and 2.0 mL of 35% sodium carbonate. The contents were further made up to 10.0 mL with distilled water and the mixture was shaken thoroughly and allowed to stand still for 30 min. Absorbance of the blue color developed was read at 700 nm against the reagent blank using PerkinElmer Lambda-35 UV–Visible spectrophotometer. Quantum of polyphenols present in tea leaves was computed using the standard calibration curve derived from known concentrations (10 to 50 ppm) of gallic acid (Sigma chemicals Private Limited, Bangalore) and the results were expressed as per cent gallic acid equivalents^9^.

### Determination of catechins

One mL of the alcoholic extract was diluted to 50 mL with distilled water. To the 2.0 mL of the diluted extract, 6.5 mL of ice cold vanillin (1% vanillin in 70% sulphuric acid) was added slowly to avoid immediate colour development. The contents were made up to 10 mL with distilled water, shaken well and allowed to stand still for 15 min for completion of reaction. Absorbance of the orange colour developed was read at 500 nm against the reagent blank in PerkinElmer Lambda-35 UV–Visible spectrophotometer. Amount of catechins present in tea leaves were calculated using the standard calibration curve computed with the values obtained against known concentrations (10 to 50 ppm) of ( +) catechin (Sigma Chemicals Private Limited, Bangalore) and the results were expressed as per cent catechin equivalents^10^.

### Estimation of DPPH radical scavenging activity

Briefly, a 1 mM solution of DPPH radical solution in ethanol was prepared, and then 1 mL of this solution was mixed with 3 mL of extract solution in ethanol containing 50–500 μg of dried extract; the mixture was then vortexed vigorously and left for 30 min at room temperature in the dark and the absorbance was measured at 517 nm with a spectrophotometer and is calculated as DPPH Scavenging % = [(Control Absorbance – Extract Absorbance)/Control Absorbance] × 100. For control 3 mL of distilled water was added to 1 mL of 1 mM solution of DPPH radical solution and the rest of the procedures remain the same^9^.

### Analysis methods for macro and micro nutrient content, instrument and quality assurance

Total N was calculated using the Kjeldahl method^11^ and the distillation was done using Gerhardt equipment. Wet digestion of dried and ground samples in a 9:4 mixture of nitric acid and perchloric acid resulted in the determination of macro (P, K, Mg, Ca, and Na) and micro elements (Cu, Fe, Mn, and Zn)^12^. Using a GBC model UV–Visible Spectrophotometer, P was determined spectrophotometrically in the diluted digests using the ammonium molybdate method following its interaction with ascorbic acid^12^. By using a flame photometer (Sherwood 410) to measure potassium and sodium in digested samples, and an atomic absorption spectrophotometer to estimate magnesium, calcium, iron, copper, manganese, and zinc. (GBC 908 AA). After creating a standard curve by feeding the certified standard solutions (made by Merck,traceable to NIST) for each nutrient, the readings were collected using the corresponding hollow cathode lamps. The flame photometer has the following specifications: 0.05 ppm sensitivity, 0% drift, better than 2% mid-range linearity, less than 20 ppb limit of detection, variable aspiration rate of 2–6 ml/min, and interference of less than 0.5%. The typical wavelengths of sodium and potassium are 589 nm and 766 nm, respectively, and the emission colors are yellow and red. As a benchmark, sodium chloride and potassium chloride are used. As a solvent, distilled water is employed. Double beam optics and programmable flame control are included in the specifications for the atomic absorption spectrophotometer with a graphite furnace. Air-acetylene/nitrous oxide burner is used for the flame, which has a 5 ppm sensitivity, automatic wavelength and slit setting, and Hyper-Pulse background correction, which ensures more precise correction of quick background signals. Detector using a photomultiplier tube with the entire 175–900 nm wavelength range. For the majority of elements, the detection limits for the graphite furnace are in the ppb range. The reduction of interference issues is achieved by the improvement of instruments.

### Statistical analysis

The experiment was a completely randomized block design with four replications. Data were subjected to analysis of variance (ANOVA) and means were separated by Duncan multiple range test at P < 0.05 significant level^13^.

## Results and discussion

### Seasonal variation on total polyphenols and catechins

The total polyphenolic and catechin content values of young shoots belong to five different cultivars is presented in Table 2. Significant differences on total polyphenolic and catechin contents were attained in various tea cultivars at different harvest periods. A variation in polyphenol content was observed in fresh shoots due to variation of seasons. It was found to be maximum during summer followed by premonsoon seasons in all the cultivars studied. It also indicated that polyphenol production was the lowest during monsoon, when there was heavy rainfall. Additionally, there were less sunshine hours at this time, which may have contributed to the tea shoots' accumulation of fewer polyphenols^14^. This explains why there is a substantially lower percentage of polyphenols in shady tea flushes. Based on this knowledge, it is possible that in addition to temperature effects, day length and sunshine effects may contribute to changes in total phenolic levels between fresh tea shoots picked in various months in south India. To clarify how day length and sunlight exposure related to the UV index induce the production of total phenolics, more research is needed. The phenolic composition of tea shoots previously changed significantly in the field due to seasonal, genetic, and agronomic factors that cause seasonal fluctuations, which could include one or more of the following environmental conditions: day length, sunlight, and/or temperature^14^. Since low-density lipoproteins can be oxidised to create atherosclerosis and coronary heart disease, tea shoots' higher amount of total phenolics is crucial for lowering the risk of these conditions. The data would suggest that there is a potential for producing premium black tea throughout the summer in south India, and these observations are consistent with Muthumani's findings^15^. According to Benti et al.^14^, different clones have different levels of phenolics in green tea shoots. As a subset of polyphenols, catechins exhibited the same tendency as polyphenols (Table 2).

**Table 2:** Variation in antioxidant activity and total phenolic content of tea shoots according to season.

Cultivars used	Harvesting period	Scavenging of DPPH radicals (%)	Quantity of phenol (%)	Overall catechins (%)
UPASI-3	Summer	66.32	32.10	21.58
	Premonsoon	64.91	29.02	19.04
	Monsoon	62.17	27.81	17.19
	Winter	64.26	28.10	18.54
	Mean	64.42	29.26	19.09
UPASI-9	Summer	65.29	31.60	20.10
	Premonsoon	63.57	28.56	18.64
	Monsoon	61.07	27.54	16.93
	Winter	62.81	27.92	18.05
	Mean	63.19	28.91	18.43
UPASI-17	Summer	64.87	29.95	21.47
	Premonsoon	62.21	28.42	18.17
	Monsoon	60.11	27.52	16.57
	Winter	61.24	27.69	17.55
	Mean	62.11	28.40	18.44
Assam seedlings	Summer	64.29	29.92	19.39
	Premonsoon	62.14	28.30	17.60
	Monsoon	60.03	25.21	16.11
	Winter	60.90	26.92	17.20
	Mean	61.84	27.59	17.58
TRI-2043	Summer	63.80	29.51	18.83
	Premonsoon	61.18	26.54	16.81
	Monsoon	57.31	22.86	15.71
	Winter	58.13	23.25	15.92
	Mean	60.11	25.54	16.82
Statistical significance (C.D. @ P = 0.05)				
Between cultivars (C)		1.00	0.69	0.64
Between seasons (S)		1.06	0.62	0.58
Interactions (C × S)		2.11	1.39	1.15

### Seasonal variation on antioxidant activity

Tea shot age, variety, season, and climate all affect the composition of the shoot^16^. Since reactive oxygen species (ROS) are thought to play a role in a number of diseases, including cancer, cardiovascular, and neurological disorders, tea and the ingredient catechins have been studied in these conditions. Data on antioxidant activity acquired at various harvest periods in tea cultivars and statistically evaluated are shown in Table 2, along with differences across seasons and cultivars. For all cultivars, antioxidant activity as DPPH radical scavenging activity ranged from 57.31% (TRI-2043) to 66.32% (UPASI-3), with summer harvesting of tea shoots showing the highest levels and monsoon harvesting the lowest. Previous research on tea indicated that the antioxidant activity of various tea products in various solvents ranged from 56 to 83%^17^. According to Pruteanu et al.^6^, goods that are frequently ingested, including tea, coffee, and chocolate, have significant antioxidant qualities. The aforementioned findings made it very evident that tea shoots had strong antioxidant activity. The significant variation in antioxidant activity among tea shoots at various harvest times is thought to be a result of changing ecological conditions. Numerous epidemiological research and experiments on animals have demonstrated that green tea can offer defence against a number of malignancies, including those of the skin, breast, prostate, and lung^18^.

### Seasonal variation on macro nutrients

Table 3 displays the macronutrient composition of five different cultivars' tea shoots at various times of the year. On the basis of the mineral content of all tea cultivars, differences between the various harvest periods were seen. Nitrogen is an economically important nutrient of the tea bush where it contains as much as 3–5% in two leaves and bud used for manufacture^19^. Higher nitrogen content influences the biochemical constituents to a greater extent and it contributes to N-containing compounds, amino acids, etc., which in turn influence the quality of commercial black teas. Seasonal influence was very significant where ‘N’ was as high as in summer followed by winter, premonsoon, winter and monsoon periods. Higher amount of nitrogen recorded during summer periods could be due to the foliar application of urea coupled with muriate of potash to impart drought tolerance in young and mature tea fields^20^. Tea plantations in south India adopt the recommended schedule of foliar application (macro and micro nutrients) in order to protect the plants from soil moisture stress and to enhance the productivity^21^. Phosphorous plays a key role in DNA synthesis and is indispensable for growth,it is an important element required for new wood formation on pruning and roots growth. However, harvested crop shoots contain 0.20–0.25% ‘P’ on dry weight basis^22^ .As far as ‘P’ is concerned, ‘P’ content registered higher in summer and least during monsoon. Maximum phosphorous content recorded in tea leaves during summer periods could be attributed to rock phosphorous application along with citric acid exerted in the summer months^23^. In order to improve both yield and quality of tea, application of K fertilisers are very important^24^. Potassium content in the shoots was in the order of winter > summer > premonsoon > monsoon. Interestingly all the seasons registered leaf ‘N’’K’ ratio of 2:1 irrespective of the cultivars. In general, relatively higher amount of nitrogen followed by potassium and phosphorus was observed whereas in the case of secondary nutrients, calcium edge over sodium content in the crop shoots. Among the nutrients, Mg ranked third in the order, after N and K in terms of its importance in the metabolic pathway of the plants and it is the only mineral constituent present in the nucleus of the chlorophyll molecule which participates in the process of photosynthesis. Magnesium content of harvestable shoots varies between 0.20 and 0.30%^23^ and it was in the order of winter, premonsoon, monsoon and summer respectively. Higher amount of magnesium was observed in winter manufactured teas could be due to application of magnesium sulphate along with muriate of potash (MOP) during winter.

**Table 3:** Seasonal variation of macro nutrients content of tea shoots.

Cultivars used	Harvesting period	N (%)	P (%)	K (%)	Mg (%)	Ca (%)	Na (%)
UPASI-3	Summer	3.25	0.28	1.49	0.25	0.47	0.08
	Premonsoon	3.02	0.28	1.51	0.26	0.43	0.07
	Monsoon	2.81	0.22	1.47	0.25	0.41	0.06
	Winter	2.90	0.33	1.65	0.28	0.42	0.07
	Mean	3.00	0.28	1.53	0.26	0.43	0.07
UPASI-9	Summer	3.20	0.33	1.47	0.22	0.44	0.08
	Premonsoon	3.12	0.23	1.48	0.24	0.41	0.07
	Monsoon	2.75	0.20	1.36	0.22	0.39	0.07
	Winter	2.82	0.23	1.56	0.25	0.39	0.06
	Mean	2.97	0.25	1.47	0.23	0.41	0.07
UPASI-17	Summer	3.21	0.36	1.50	0.23	0.38	0.08
	Premonsoon	3.04	0.25	1.45	0.22	0.35	0.06
	Monsoon	2.74	0.27	1.32	0.23	0.32	0.06
	Winter	2.81	0.25	1.54	0.26	0.35	0.08
	Mean	2.95	0.28	1.45	0.24	0.35	0.07
Assam seedlings	Summer	3.09	0.35	1.52	0.21	0.34	0.07
	Premonsoon	2.74	0.26	1.39	0.21	0.32	0.07
	Monsoon	2.71	0.27	1.30	0.21	0.30	0.07
	Winter	2.89	0.29	1.67	0.24	0.31	0.08
	Mean	2.86	0.29	1.47	0.22	0.32	0.07
TRI-2043	Summer	2.94	0.30	1.37	0.20	0.33	0.08
	Premonsoon	2.66	0.29	1.30	0.21	0.30	0.08
	Monsoon	2.30	0.27	1.18	0.22	0.28	0.07
	Winter	2.46	0.32	1.42	0.24	0.29	0.08
	Mean	2.59	0.30	1.32	0.22	0.30	0.07
Statistical significance (C.D. @ P = 0.05)							
Between cultivars (C)		0.04	0.01	0.02	0.01	0.01	0.01
Between seasons (S)		0.04	0.01	0.02	0.01	0.01	0.01
Interactions (C x S)		0.08	0.02	0.04	0.02	0.02	0.02

*N* nitrogen, *P* phosphorous, *K* potassium, *Mg* magnesium, *Ca* calcium, *Na* sodium, *UPASI* United Planter’s Association of Southern India.

### Seasonal variation on micro nutrients

Among the clones, UPASI-3 accumulated higher amount of Cu, Mn and Zn except Fe (Table 4). Quantum of occurrence of micro nutrients followed a distinct order where Mn scored first position and forced down the other elements in subsequent positions. Except ‘Cu’, other micro nutrients are enriched during summer followed by other seasons. In order to protect tea plants from the fungus *Exobasidium vexans* Massae, which causes blister blight, contact fungicide copper oxychloride is applied in conjunction with systemic fungicides^25^. Copper requirements are anticipated to be satisfied by the foliar application of copper oxychloride^26^. It deserves close attention and is of particular importance in tea biochemistry because the polyphenol oxidase (PPO) contains this mineral as a central metal atom. Clones of tea exhibited selectivity in the uptake and accumulation of micronutrients, leading to their dispersion in different directions. The redox characteristics of iron make it a necessary element for plant metabolism. It is crucial to basic biological functions like photosynthesis, respiration, nitrogen fixation, and DNA synthesis^27^. During the present study, the concentration of iron content in various seasons was ranged between 127.82 mg/kg (monsoon and TRI-2043) and 167.13 mg/kg (summer and UPASI-3). Lower amount of manganese was observed during monsoon when the micronutrients application is not recommended in south Indian tea plantations which could be resulted in lower leaf manganese concentration. Zinc takes part in the synthesis of plant growth regulating substances like indole acetic acid and auxins which regulate the growth and development^28^ and maintain the higher productivity^20^. Among the seasons, zinc was significantly higher during summer periods compared to rest of the seasons. This may be attributed to foliar application of zinc sulphate to reap the early crop^21^. Present study clearly indicated that the nutrient content of tea shoots depends on the cultural operations followed by prevailing climatic variables besides the genetic potential of the cultivars. It is noted that minerals are crucial for both plant and human nutrition. It is possible to find potassium, a mineral important for regulating the salt balance in human tissue. Tea shoots contain high concentrations of zinc, a trace mineral that is crucial for the immune system's normal operation. Five distinct cultivars have relatively high levels of calcium, a mineral that is crucial for bone health and function.

**Table 4:** Seasonal variation of micro nutrient contents of tea shoots.

Cultivars used	Harvesting period	Cu (ppm)	Fe (ppm)	Mn (ppm)	Zn (ppm)
UPASI-3	Summer	17.29	167.13	323.44	42.28
	Premonsoon	18.39	154.77	312.25	41.83
	Monsoon	21.21	141.96	304.65	38.31
	Winter	20.02	151.06	312.02	39.17
	Mean	19.23	153.73	313.09	40.40
UPASI-9	Summer	16.77	175.38	295.12	34.34
	Premonsoon	17.74	162.32	279.38	32.23
	Monsoon	20.62	159.36	267.85	29.71
	Winter	19.35	164.09	271.10	30.49
	Mean	18.62	165.29	278.36	31.69
UPASI-17	Summer	14.70	163.25	284.12	35.30
	Premonsoon	15.72	149.21	275.34	32.95
	Monsoon	18.47	152.38	270.16	28.30
	Winter	17.26	162.01	275.35	29.26
	Mean	16.54	156.71	276.24	31.46
Assam seedlings	Summer	14.69	145.21	268.97	32.20
	Premonsoon	15.29	135.08	257.66	30.07
	Monsoon	17.97	133.21	245.12	27.93
	Winter	16.98	141.02	257.12	28.16
	Mean	16.23	138.63	257.22	29.59
TRI-2043	Summer	11.56	139.75	251.08	30.84
	Premonsoon	13.21	130.45	249.10	29.68
	Monsoon	13.29	127.82	231.31	27.50
	Winter	11.59	134.06	241.51	28.47
	Mean	12.41	133.02	243.25	29.12
Statistical significance (C.D. @ P = 0.05)					
Between cultivars (C)		0.02	0.05	0.01	0.03
Between seasons (S)		0.02	0.04	0.01	0.03
Interactions (C × S)		0.04	0.09	0.02	0.06

## Conclusion

Tea shoots might be assumed to be a useful source given their high antioxidant capacity and beneficial nutrient content. Crop shoots harvested during the summer months produced higher levels of polyphenol and total catechins, which are predicted to result in tea of superior quality. The antioxidant activity exhibited higher values in crop shoots collected in the summer months and the lowest value in monsoon period. Regardless of cultivar, summertime levels of nitrogen, phosphorous, calcium, and salt were higher than wintertime levels, while wintertime levels of potassium and magnesium were superior. Important data from this study showed that while zinc, iron, and manganese levels were greater in the summer, copper levels were higher during the monsoon. This study discovered that the harvest season has a substantial impact on the phenolic profiles, macro and micronutrient profiles, and antioxidant capacity of south Indian black teas. However, more research is needed to better understand the role of individual and synergistic environmental factors on observed variability.

## Data Availability

The datasets used and analyzed during the current study are available from the corresponding author on request.
